# The National Institute of Health Research (NIHR) Collaboration for Leadership in Applied Health Research and Care (CLAHRC) for Leicestershire, Northamptonshire and Rutland (LNR): a programme protocol

**DOI:** 10.1186/1748-5908-4-72

**Published:** 2009-11-12

**Authors:** Richard Baker, Noelle Robertson, Stephen Rogers, Melanie Davies, Nigel Brunskill, Kamlesh Khunti, Michael Steiner, Martin Williams, Paul Sinfield

**Affiliations:** 1 Department of Health Sciences, University of Leicester, Leicester, UK; 2 School of Psychology, University of Leicester, Leicester, UK; 3 Northamptonshire Primary Care Trust, Northampton, UK; 4 Department of Cardiovascular Sciences, University of Leicester, Leicester, UK; 5 Department of Infection, Immunity and Inflammation, University of Leicester, Leicester, UK; 6 University Hospitals of Leicester NHS Trust, Leicester, UK; 7 NIHR CLAHRC for LNR, UK

## Abstract

**Background:**

In October 2008, the National Institute for Health Research launched nine new research projects to develop and investigate methods of translating research evidence into practice. Given the title Collaborations for Leadership in Applied Health Research and Care (CLAHRC), all involve collaboration between one or more universities and the local health service, but they are adopting different approaches to achieve translation.

**Methods:**

The translation and implementation programme of this CLAHRC has been built around a pragmatic framework for undertaking research to address live concerns in the delivery of care, in partnership with the managers, practitioners, and patients of the provider organisations of the CLAHRC. Focused on long-term conditions, the constituent research themes are prevention, early detection, self-management, rehabilitation, and implementation. Individual studies have various designs, and include both randomised trials of new ways to deliver care and qualitative studies of, for example, means of identifying barriers to research translation. A mix of methods will be used to evaluate the CLAHRC as a whole, including use of public health indicators, social research methods, and health economics.

**Discussion:**

This paper describes one of the nine collaborations, that of Leicestershire, Northamptonshire, and Rutland. Drawing a distinction between translation as an organising principle for healthcare providers and implementation as a discrete activity, this collaboration is built on a substantial programme of applied research intended to create both research generation and research use capacity in provider organisations. The collaboration in Leicestershire, Northamptonshire, and Rutland has potential to provide evidence on how partnerships between practitioners, patients, and researchers can improve the transfer of evidence into practice.

## Background

The collaborations for Leadership in Applied Health Research and Care (CLAHRCs) are new organisations funded by the National Institute for Health Research (NIHR) in England to conduct and implement applied health research, the focus being on the second translation gap, that of translating research into practice [1,2]. CLAHRCs are partnerships between a university and surrounding health service organisations, and are required to develop a model for conducting applied research and translating findings into improved outcomes. To date, nine CLAHRCs have been established, one of which is that of Leicestershire, Northamptonshire, and Rutland (LNR), a defined area in the east midlands of England with a population of around 1.6 million people. The NIHR CLAHRC for LNR involves a partnership between the University of Leicester, the postgraduate deanery, all three acute hospital trusts, all three primary care trusts, and both mental health trusts in the locality. This paper sets out the framework for translation and implementation being adopted by the CLAHRC for LNR.

The specific objectives of the CLAHRC for LNR are to: implement and evaluate a framework to increase applied research and translation in LNR; conduct applied research relating to chronic conditions of public health importance; develop and evaluate a practical approach to implementation as part of research translation; and increase local capacity in applied research. It has a combination of four inter-related applied research themes and an implementation theme (Table 1) and is focused primarily on chronic conditions of importance in the locality (diabetes, cardiovascular disease, mental health, renal disease, chronic respiratory disease, and stroke).

**Table 1:** The research themes of the NIHR CLAHRC for LNR

Themes
1. Prevention of disease

2. Early detection of disease

3. Patient education and self-management

4. Rehabilitation

5. Implementation

In the UK, a national expert group has recently reviewed the implementation research agenda [3] and among recommendations for a sustained programme of research, the group recognised the need for training programmes to increase the numbers of researchers in the field, and the embedding of researchers into the health service to both ensure that research is more responsive to the needs and context of the service and to improve the translation of the findings of implementation research into practice. The CLAHRCs, therefore, have a role to play in responding to these recommendations. In this paper, we set out the approach to translation and implementation being adopted in the NIHR CLAHRC for LNR.

## Methods

### Differentiating translation and implementation

A variety of terms has been used for the process by which evidence is adopted in practice, including implementation, translation, knowledge translation (sometimes abbreviated to KT), and knowledge mobilization; other terms referring to elements of the process include clinical effectiveness and evidence-based practice. The multiplicity of related terms can be confusing, but in the NIHR CLAHRC for LNR we concentrate on and distinguish between translation and implementation.

From the time that research is begun, several years or even decades can pass before its first impact in clinical practice [4]. A review of health research funding in the UK highlighted the need to close this gap between research and practice, and identified two contributory problems [2]. The first is the gap between the description of a new clinical intervention and initial clinical trials (sometimes referred to as the first translation gap, or T1), and the second is the gap between evaluation of new interventions in health technology assessment studies and the embedding of the new intervention in clinical practice (referred to as the second translation gap, or T2). The CLAHRC is concerned with the second translation gap; that is, getting new, effective ways of improving health into routine use.

In addition to delay in the adoption of research, there is also considerable variation between health professionals, teams, and organisations in the extent to which evidence is applied consistently in each setting with each patient. For more than three decades, healthcare organisations have attempted to reduce inappropriate variations in performance and get research into practice more effectively, but the success of these attempts has been variable. Many of the approaches used in the past have focused directly on the performance of individuals and teams, and have included educational interventions about the recommendations of guidelines (*e.g*., workshops and seminars), quality improvement interventions (*e.g*., audit and feedback), and marketing interventions (*e.g*., academic detailing). Within the CLAHRC, we refer to these approaches as implementation, an activity focused on getting research into practice. Translation, in contrast, is an overarching process in which researchers and practitioners cooperate together to improve the effectiveness of care. It may involve the adaptation of existing research findings or the conduct of new research, but it is focused on generating solutions to active problems. This process is explained further in the following paragraphs.

### The translation model

Knowledge translation is defined by the Canadian Institutes of Health Research as 'the exchange, synthesis, and ethically-sound application of knowledge--within a complex system of interactions among researchers and users--to accelerate the capture of the benefits of research for Canadians through improved health, more effective services and products, and a strengthened healthcare system' [5].

**Figure 1:**
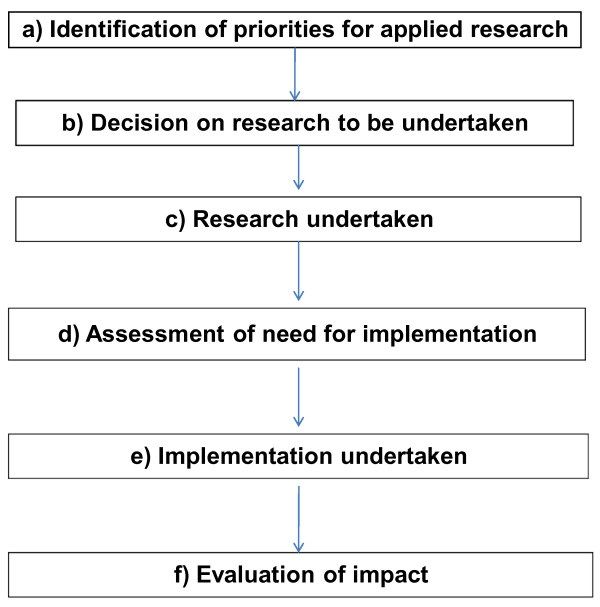
**The translation model of National Institute for Health Research Collaboration for Leadership in Applied Health Research and Care for Leicestershire, Northamptonshire and Rutland (NIHR CLAHRC for LNR)**.

The translation model being used in the CLAHRC for LNR is shown in Figure 1. The steps are: (a) identification of the priorities and needs for applied research of the health care organisation in order to improve the outcomes of its patients. (b) since there are resource and other limitations on the amount of research that can be undertaken at any one time, a decision is required on which issues will be addressed by research. Furthermore, sometimes it may be decided that new research is not required because sufficient evidence is already available, in which case implementation of the evidence would be more appropriate. (c) The required research is undertaken; if applied in nature, the research may include evidence reviews, studies of new ways of delivering services or interventions, evaluation of new interventions, or economic evaluations. The studies may be of short duration, small in scale and not require new funding, or may be longer term and require external funding. The findings should provide the evidence for decision making by the organisation, and being designed to address important questions for the organisation and its practitioners, they should be likely to be directly adopted. (d) However, sometimes, formal implementation activities may be required. An assessment of the need for implementation will be undertaken through consultation with commissioners, practitioners and patients. (e) Evidence needing systematic implementation will be taken up within the CLAHRC implementation theme. (f) Evaluation will take place, assessing the extent to which research findings have been taken up into practice and the impact on health outcomes.

The first steps (a, b) in the framework are being undertaken through discussion with decision makers in each of the eight partner trusts, and social research methods will be used to study what worked and what did not work in this process. Among research studies themselves (c) that are currently planned, several randomised trials will be undertaken of means of delivering care, for example of approaches to delivering rehabilitation in primary care. The findings will be used by those commissioning, planning or delivering care, and when necessary, formal methods of implementation will be used (the approach to implementation is described later). An example, taken from the prevention theme, concerns the identification of people at risk of depressive illness. Having established this topic as a priority for one of the mental health trusts, an intervention to identify and manage risk of depression has been developed from previous published research evidence, and following discussions with the acute care trusts, a randomised trial is planned of the modified intervention to be delivered by midwives and involving pregnant women. The findings of the trial will inform decisions on training programmes for midwives. In associated studies using non-experimental study designs, we plan to investigate the potential of management of risk of depression in another group at high risk of depression, namely people with major chronic health problems.

Our translation model has been strongly influenced by the organisational excellence model of Nutley and colleagues [6]. In this model, responsibility for research use rests largely with local service delivery organisations, and is supported by an organisational culture that is research-minded. Local adaptation of research findings will be undertaken, associated with learning within teams and the organisation, and partnerships with universities and other bodies may be used to facilitate the creation and use of knowledge. Our model is also influenced by the knowledge to action process [5] in which identification of the need for knowledge and the adaptation or tailoring of knowledge have important roles.

A further influence, taken from practice rather than theory, has been the experience of the US Veterans Health Administration (VHA), which launched a quality improvement programme as part of a major re-structuring initiative in the 1990s. The Quality Enhancement Research Initiative (QUERI) is part of the VHA's research infra-structure, and brings together in selected centres researchers, practitioners, and managers to address key healthcare issues faced by the VHA [7,8]. The QUERI process has six steps: identify high risk/volume disease/problems; identify best practices; define existing practice patterns and outcomes across the VHA, and current variation from best practices; identify and implement interventions (including performance criteria) to promote best practices; document that best practices improve outcomes; and document that outcomes are associated with improved health related quality of life.

It is difficult to be certain how much of the VHA's improvement in care [9-11] has been due to QUERI and how much to other structural changes, but reports of QUERI projects illustrate what can be achieved [12-14]. Research is an integral part of the VHA's mission, and the organisation employs its own researchers, a fact that may have facilitated the encouragement of researchers to address problems important to the organisation. While many CLAHRC researchers are based in a university, a growing number are based in the NHS Trusts, and it should be noted that the VHA also has collaborations with researchers in universities. The features of QUERI that have contributed to its impact have been reviewed by Graham and Tetroe [15]. They include an action-oriented approach with teams of managers, clinicians, patients, and researchers co-producing knowledge, against a background of transformative change with regard to how the organisation generates and uses knowledge. Systemic change of this nature, however, takes leadership, time, and persistence. Although further development of QUERI and research into ways to maximise its impact are required [16], it does suggest that the application of the organisational excellence model in healthcare deserves investigation. While it is too early to judge the success of the organisational excellence model in healthcare, the concept of bringing practitioners, managers and researchers together to address a shared goal--improvement of health of local patients--is engaging and has some initial evidence to indicate its potential [17].

### Applied health research

The applied research themes are integral to the translation model (Figure 1). They include studies designed to help providers decide whether specific clinical interventions should be translated into practice. Thus, one study will investigate the place of a new model of care to prevent progression of chronic kidney disease, another will evaluate the benefits of a scheme for early assessment of transient ischaemic attack and stroke, and a third will explore the impact of a primary care-based rehabilitation programme. These are but three examples of a programme that involves approximately 15 studies, but in addition to informing decisions about services, the applied themes serve to establish a substantial team of researchers, practitioners, and managers who are acquiring experience of using research together. As new priorities for research are identified by the trusts of the CLAHRC, these teams will be on hand to undertake or facilitate the research. As the number of staff in the trusts become involved in undertaking research studies or in applying the findings, we will be investigating the extent to which this changes the way the trusts use research in their decision making, and whether it increases their capacity to absorb and apply new research evidence, that is, whether they are developing the research minded culture of the organizational excellence model [6].

## Implementation

In our CLAHRC, implementation refers to the more established approaches to get evidence into practice that generally rest on the linear model in which research is produced by researchers, and practitioners and managers are encouraged to make use of it. Research evidence will continue to be produced by groups worldwide, and this evidence can be used to improve the health of local people, and therefore must be implemented locally. The implementation theme of the CLAHRC will employ a mix of methods, drawing on evidence of their effectiveness, informed by the reviews of the Cochrane Effective Practice and Organisation of Care (EPOC) review group. The theme will also seek to advance the methods of implementation by building on the idea of tailoring implementation methods to the barriers and enablers of change [18]. Currently, evidence for the effectiveness of this approach is equivocal [19], and research is required to determine how tailored strategies should be designed, how barriers and enablers can be most effectively identified, and which strategies should be used to address particular barriers. Implementation using methods such as these, however, can be regarded as one component of translation, as set out in our simple model. Within the implementation theme, as projects are instituted in accordance with local priorities, we will undertake associated research to develop an approach to tailoring that could be used by healthcare staff after only limited training. Our providers need efficient and practical methods that can be used routinely. Initial projects to develop aspects of this practical tailored implementation intervention are planned or underway, the first addressing the issue of implementation of guidelines on obesity in primary care. This study will compare tailoring undertaken by two independent groups in order to identify some of the training needed by healthcare staff to enable them to tailor implementation to barriers and enablers. In due course, we aim to undertake a randomised trial of the practical tailored intervention.

## Discussion

The creation of the nine CLAHRCs in England constitutes a major investment in research into how evidence can be translated into practice, and demonstrates the importance now placed on this issue by the NIHR in England. In the coming years, much will be learned about translation in the context of a publicly funded health service that is required to comply with national policy. In this paper, we have described the particular approach that is being applied in one CLAHRC. Underpinned by a substantial programme of applied research designed to increase the capacity of healthcare trusts to apply evidence, the approach makes a distinction between translation and implementation. While implementation is regarded as the use of more established interventions within a more linear framework for understanding the process of getting research into practice, translation is regarded as a new, broader, collaborative approach that brings clinicians, researchers, patients, and managers together to improve care. Various evaluation studies of the NIHR CLARHC for LNR are planned, and other studies will investigate and compare the activities of all the CLAHRCs. The CLAHRCs have been established for a period of five years in the first instance. This is a short timeframe if major change is to be demonstrated, but whether or not CLAHRCs have a positive impact on translation within the time allowed, it should be possible to develop a better understanding of how healthcare organisations can work with researchers to translate knowledge into better healthcare.

## Competing interests

The authors declare that they have no competing interests.

## Authors' contributions

The model was originally developed by RB, MW, MD, NB, KK, and MS. The model was further developed by NR, SR and PS. The first draft of the paper was prepared by RB, and then all the authors contributed to its development and completion.
